# Malignant Glandular Triton Tumor Arising in the Radial Nerve with Prolonged Survival: A Case Report and Review of the Literature

**DOI:** 10.1155/2021/4614185

**Published:** 2021-03-18

**Authors:** Batool M. AlAli, Samir S. Amr

**Affiliations:** ^1^Department of Pathology and Laboratory Medicine, King Fahad Specialist Hospital, Dammam, Saudi Arabia; ^2^Department of Pathology and Laboratory Medicine, Istishari Hospital, Amman, Jordan

## Abstract

Divergent differentiation is a well-known phenomenon in malignant peripheral nerve sheath tumors (MPNST) which occurs approximately in 15% of these tumors, usually towards mesenchymal elements. Differentiation towards epithelial components, however, is quite uncommon, and even exceptionally rare is concomitant mesenchymal and glandular differentiation. To our knowledge, only 14 cases of MPNST with both mesenchymal (rhabdomyoblastic) and glandular differentiation had been reported, and only two of these tumors had frankly malignant glandular components. Herein, we report the third such case. A 26-year-old male, without any of the stigmata of NF1, presented with a 2-year history of pain in his left shoulder and an elbow swelling of six-month duration. The tumor was initially diagnosed clinically as a neurofibroma at a local hospital. The patient underwent excision of the mass there, and pathological examination at that hospital showed the tumor to be MPNST. Six months later, the patient was referred to our hospital, a tertiary care medical center, with recurrent swelling at the same location. Histopathological material from the referral hospital was reviewed, and the tumor was diagnosed as MPNST with rhabdomyoblastic differentiation or malignant triton tumor (MTT) that contained in addition foci of malignant glandular epithelium. The patient refused any surgical intervention. He received three cycles of chemotherapy followed by radiotherapy with excellent response and marked reduction in the size of the tumor. The patient had prolonged survival for 10 years following the initial resection of the tumor.

## 1. Introduction

Malignant peripheral nerve sheath tumors (MPNST), which were known in the past as malignant schwannoma, neurofibrosarcoma, or neurogenic sarcoma, are soft tissue sarcomas with evidence of endoneurium and perineurium differentiation. Peripheral nerves are the origin of approximately half of the cases, and neurofibromas are the origin of the other half especially in the setting of neurofibromatosis type 1 (NF1) (von Recklinghausen disease).

Divergent differentiation which is a well-known phenomenon in MPNST occurs approximately in 15% of the cases and usually toward mesenchymal elements with either rhabdomyoblastic, chondroid, myoid, or osteoid elements [1]. MPNST with rhabdomyosarcomatous was first described as a tumor entity by Masson in 1932 [2] and was reported for the first time as “malignant triton tumor” (MTT) by Woodruff et al. in 1973 [3]. Differentiation towards epithelial components is quite uncommon with the first glandular malignant schwannomas (MPNST with glandular differentiation) reported by Garré in 1892 [4]. To the best of our knowledge, only fourteen cases of MPNST with both rhabdomyoblastic and glandular differentiation had been reported, and only two of these tumors had frankly malignant glandular components, and two other cases showed glands with nuclei featuring atypia. Herein, we report the third case of malignant triton tumor with malignant glandular differentiation stressing the ability of MPNSTs in having both mesenchymal (rhabdomyoblastic) and epithelial (malignant glandular) divergent differentiation occurring simultaneously, with a review of the relevant literature.

## 2. Case Presentation

A 26-year-old male, not known to have NF1, presented with a 2-year history of pain in his left shoulder and an elbow swelling of six-month duration. He was initially diagnosed as having neurofibroma on the basis of tru-cut core biopsy of the mass done at a peripheral hospital. He underwent an excision of the tumor in October 2010 at another facility, and the histopathological diagnosis made there was MPNST. Six months later, the patient was referred to our hospital with a recurrent mass at the site of the previous excision, associated with pain that awakened the patient from sleep. On examination, there was a large surgical scar on the left elbow about 12 cm. in length and an underlying lobulated swelling extending proximally and distally with left radial nerve palsy (wrist drop).

**Figure 1:**
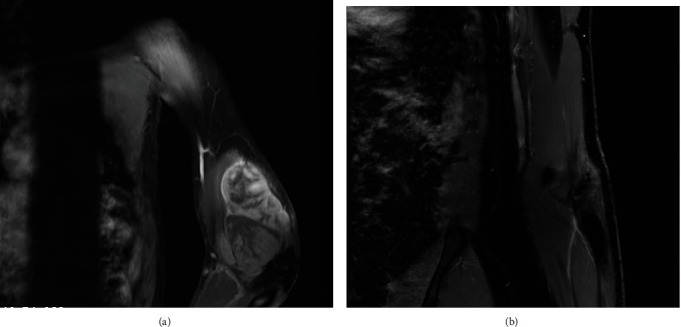
(a) This MRI image was taken at first presentation in 2011. It showed a large lobulated heterogeneous mass extending from the middle of the left arm down to the middle of forearm, measuring 29 × 9 × 9 cm. (b) The last MRI imaging done dated on January 2018. No evidence of circumscribed mass lesion or abnormal enhancement to suggest tumor recurrence. No new lesions identified.

Magnetic resonance imaging (MRI) scans showed a large lobulated heterogeneous mass extending from the middle of the left arm down to the middle of forearm, measuring 29 × 9 × 9 cm. The lesion is predominantly in the volar aspect of both the left arm and the forearm, crossing the elbow joint, with no definite evidence of intra-articular or bony involvement. The radial neurovascular bundle cannot be separated from the lesion (Figure 1(a)). Two small subcentimetric pulmonary nodules at the upper lobe of the left lung of uncertain significance were seen.

**Figure 2:**
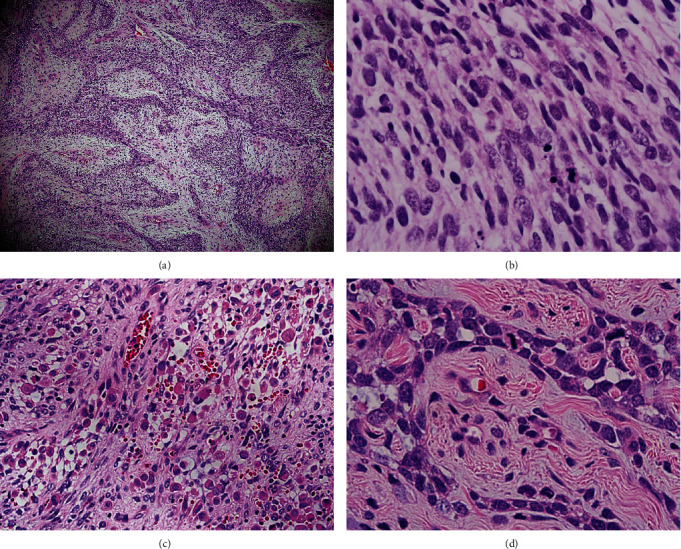
(a) Photomicrograph showing a neoplasm predominantly composed of spindle cells arranged in interlacing fascicles of alternating hypercellular and hypocellular areas (marbleization). H&E ×40. (b) The nuclei are pleomorphic, hyperchromatic, and mitotically active. H&E ×200. (c) Focus within the tumor shows rhabdomyoblastic differentiation. H&E ×100. (d) The glandular components seen lined by malignant columnar epithelial cells. H&E ×400.

Histopathology slides from the referring hospital were reviewed, The tumor was predominantly composed of spindle cells arranged in interlacing fascicles, with a pattern of alternating dense and hypodense areas giving the so-called marbleized appearance (Figure 2(a)), with pleomorphic, hyperchromatic nuclei, and increased mitotic activity (Figure 2(b)). Large areas of necrosis were present with perivascular sparing of malignant tumor cells, with the so-called survival phenomenon noted. There were several foci of rhabdomyoblastic differentiation featuring large rhabdomyoblasts with abundant eosinophilic cytoplasm (Figure 2(c)). Some fields exhibit glandular differentiation featuring glands lined by malignant columnar epithelial cells, with mitotic figures and large pleomorphic nuclei (Figure 2(d)).

**Figure 3:**
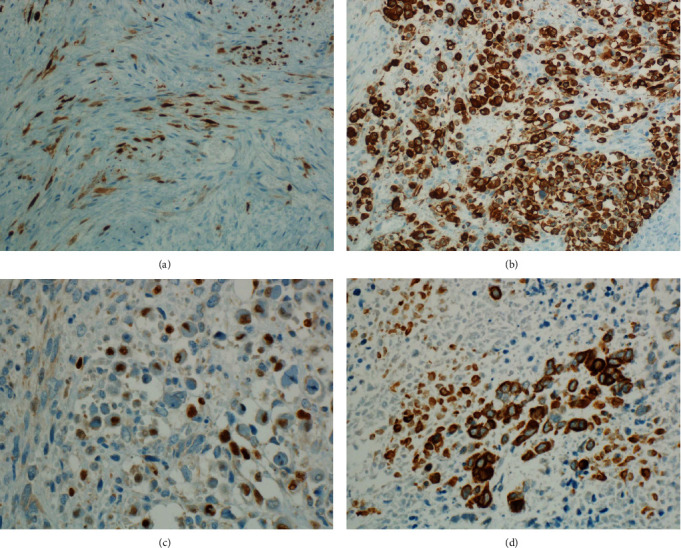
(a) Immunohistochemical (IHC) staining for S100 protein shows patchy nuclear and cytoplasmic expression. (b) Rhabdomyoblastic cells are strongly positive for desmin. (c) Within the foci of rhabdomyoblastic differentiation, there are cells showing nuclear myogenin expression. (d) The glandular components are highlighted by pan-cytokeratin positivity.

Immunohistochemically, the spindle cell component showed patchy nuclear and cytoplasmic S100 protein expression (Figure 3(a)), while the rhabdoid cells were strongly positive for desmin (Figure 3(b)), and within the desmin-positive areas, there were cells showing nuclear myogenin expression (Figure 3(c)). The malignant epithelial cells of the glandular component were positive for pan-cytokeratin (pan-CK) (Figure 3(d)), but were negative for chromogranin and synaptophysin.

Our revised diagnosis was MPNST with rhabdomyoblastic differentiation (MTT) that contained foci of malignant glandular epithelium.

The patient refused any surgical intervention which would require disarticulation. He received three cycles of chemotherapy (doxorubicin and ifosfamide) followed by radiation therapy 60 Gray (Gy) units over 30 fractions that ended in 2011, with marked improvement of his lesions (Figure 1(b)). At the time of reporting this case in January 2021, the patient had no evidence of clinical recurrence or residual disease or signs of metastasis.

## 3. Discussion

When Woodruff et al. reported the first case of “malignant triton tumor,” he proposed three criteria for the diagnosis of this tumor entity: tumor arising within anatomic location of a major nerve or neurofibroma in a patient with NF1, immunohistochemical and/or ultrastructural evidence of Schwann cell differentiation, and rhabdomyoblasts arising within the body of the tumor [3].

Although our patient was not known to have NF1, the distribution of the lesion along the left radial nerve, the immunohistochemical evidence of Schwann cell differentiation, and the presence of rhabdomyoblasts within the tumor led to the diagnosis of MTT.

The presence of benign or malignant glands in association with MTT is exceedingly rare. We did an extensive review of the literature utilizing search for terms “MPNST with divergent differentiation,” “MPNST with rhabdomyoblastic differentiation,” “malignant Triton tumor,” and “malignant glandular triton tumor.” There were only 14 cases of MPNST with both rhabdomyoblastic (MTT) and glandular differentiation having been reported (Table 1). Twelve of these cases showed benign epithelial elements, and only two had frankly malignant glandular components. Two cases showed glands with atypia [5–17].

Review of the reported case of malignant triton tumor (MTT) with benign, atypical, or malignant glandular differentiation (Table 1) revealed that there were six patients who had surgery only with available follow-up data who were dead of their disease at 3 months (MTT with chondrosarcoma, osteosarcoma, and benign glands), 4 months (MTT with benign glands), 6 months (MTT and malignant glands), and 19 months (MTT with benign glands). One patient who had mediastinal triton tumor with benign glands which was resected surgically was alive with disease with brain metastasis at 24 months, and one patient had tumor recurrence after 5 years. This patient with the long-term survival had MTT and benign glands.

Regarding the four patients who had surgery with radiotherapy and chemotherapy with follow-up data, three of them were dead of their disease: the first at 15 months (MTT with malignant glands), the second at 18 months (MTT with atypical glands), and the third at 33 months (MTT with markedly atypical gland). The fourth patient had recurrence after 10 months without metastasis (MTT with benign glands, lipoblasts, and ganglion cells).

Two patients had surgical resection but no follow-up data. Another two patients had no details regarding their management or follow-up information.

It seems that patients who were managed by surgery followed by chemotherapy and radiotherapy had slightly better survival (average was 22 months), when compared with those who had surgery alone (average survival was 8 months). Not included are patients with no documented point of death.

Six patients had NF1, and only four of them had follow-up data. They were dead of their disease at 3, 4, 18, and 19 months. Five patients did not have stigmata of NF1. All of them had follow-up data. Three were dead of their disease at 6, 15, and 33 months. Two patients had recurrence of their tumor at 24 months and 60 months (5 years).

It is difficult to speculate on the length of survival of the patients who had malignant glandular triton tumor with and without NF1 gene mutation on a small number of cases. However, from the small number of available cases, it seems that the patients without the NF1 mutation fair better with survival up to five years. Our patient did not have the mutation and survived up to ten years.

Our patient did not have NF1, and his tumor showed MTT with malignant glands. He underwent resection, but the tumor recurred and he received both radiotherapy and chemotherapy with long-term survival of ten years.

The prognosis of patients with MTT is poor, and it is worse than classical MPNST, with a 10–20% 5-year survival rate for MTT and 34–44% for MPNST [18]. However, the present patient had been recurrence-free for 10 years after the initial surgical excision that was followed by chemotherapy and radiation therapy, and he is still checked annually with CT scan follow-up. As shown in Table 1, there are no previous reports of MTT with survival of more than 5 years.

Although there are some circumstances where the diagnosis of MPNST should be considered, as stated in the Woodruff criteria listed above, the histological diagnosis of MPNST is often difficult especially in sporadic setting. Since MTT with glandular differentiation is an extremely rare malignancy, other morphology mimickers should be considered such as biphasic synovial sarcoma, sarcomatoid carcinoma, and metastasis of adenocarcinoma into peripheral nerve sheath tumor. Neural markers S100, SOX10, and GFAP are positive in 40-50%, 30%, and 30%, respectively, in cases of MPNSTs. This emphasizes the need for more specific diagnostic tools [19]. H3K27 trimethylation loss by immunohistochemistry was found to be a diagnostic marker for MPNSTs, observed in 34% of cases in a series of 162 MPNSTs. However, this marker was retained in all 97 neurofibromas and was retained also in 43 out of 44 schwannomas stained for this marker. Interestingly, loss of this marker was observed in 60% (9/15) cases of synovial sarcoma cases stained, making this marker not suitable to distinguish between MPNST and synovial sarcoma, which is one of its mimickers. In addition, loss of H3K27me3 was found to be related to poorer survival in MPNSTs [20].

Hirbe et al. in a study of BRAFV600E mutation in sporadic and neurofibromatosis type 1-related MPNST utilizing immunohistochemical technique found that this mutation was observed in 20% (5/25) of sporadic MPNSTs. In these BRAFV600E-positive MPNSTs, 90%–100% of the tumor cells were BRAFV600E-immunoreactive, suggesting that this mutation could be a primary driver of malignancy and not merely a mutation found in a subset of tumor cells. BRAFV600E mutation was not observed in benign neurofibromas (0/11 tumors), implicating BRAF mutation in malignant progression rather than in neurofibroma tumorigenesis. Only one NF1-associated MPNST harbored a BRAFV600E mutation (1/37; 2.7%) [21].

In a recent review of the genetics and genomics of MPNSTs, Lemberg et al. reported that genetic sequencing particularly in NF1-associated MPNST converged on a set of four common genetic changes that occur in most MPNST, including neurofibromin 1 (NF1), CDKN2A, TP53, and members of polycomb repressor complex 2 (PRC2) [22].

In another review of genetics of MPNSTs, Pemov et al. emphasized the same findings, namely, that somatic changes in NF1, CDKN2A/B, and PRC2 are found in most MPNSTs regardless of their etiology. Genomic studies identified previously unrecognized critical involvement of PRC2 core components SUZ12 and EED in transition to malignancy. MPNSTs carry a relatively low burden of single nucleotide variants but consistently display a high number of structural copy number variants [23].

MPNSTs have a capacity for divergent differentiation surpassed only by germ cell tumors. It is postulated that the neoplastic cells do not induce differentiation but do themselves differentiate to express a rhabdomyoblastic phenotype. Cranial neural crest cells are known to give rise to mesenchymal structures during the development of the cranium. On the basis of this role of neural crest cells during development, it is accepted that mesenchymal differentiation is part of the repertoire of those cells that give rise to MPNSTs [24]. Proliferation of more than one subclone of neoplastic cells in MPNST had been referred to as MPNST with pluridirectional differentiation [25]. Usually, mesenchymal components of MPNSTs with pluridirectional differentiation (skeletal muscle, cartilage, bone, and blood vessels) are malignant, but epithelial cells usually show benign features [25]. The present case exhibited malignant glandular elements.

In a series of 116 MPNSTs from Mayo Clinic, 17 cases (14.7%) exhibit divergent differentiation. Heterologous mesenchymal components were present both singly and in combination. The most common was rhabdomyoblastic differentiation (MTT) seen in 10 cases. Elements of osteosarcoma and chondrosarcoma were present in six tumors each. Epithelial differentiation was observed in two tumors only. In one, both malignant glandular and squamous epithelial elements were seen. However, no combination of rhabdomyoblastic and glandular differentiation was observed in this series [1]. In another series of 160 cases of MPNSTs from France, there were 22 cases of MTT (17.7%), but no other divergent differentiation was reported [26].

In a comprehensive review of 84 cases of MTT reported between 1932 and 1994, Woodruff and Perino demonstrated that 57% of the cases had NF1. The mean age was 31 years for those patients with NF1 and 37 years for those without NF1. Thirteen tumors contained other heterologous elements and were designated by the authors as pluridirectional MTT. In 7 of those 13 tumors, the additional element or one of the additional elements was glandular epithelium. 86% of patients with glandular pluridirectional MTT had NF1 [27].

MPNSTs of the radial nerve had been reported in single case reports or in review articles. In 1935, Arthur Purdy Stout, the legendary American surgical pathologist and a prolific author on soft tissue tumors, reviewed 137 tumors in 100 patients with NF1 and 29 patients without NF, including 8 cases from his lab. There were five tumors arising from the radial nerve and its branches [28]. He reported in 1942 an additional case of MPNST with epithelial differentiation involving the radial nerve in the antecubital fossa in a 35-year-old male. The patient developed lung metastases within one year [29]. Hackel reported in 1934 a case of MPNST of the radial nerve with glandular differentiation in a 37-year-old woman [30]. A unique case of MPNST with angiosarcomatous divergent differentiation was reported by Macaulay in the right radial nerve of an 18-year-old male with NF1. The tumor measured 15 × 5 × 5 cm and had metastasized to the lungs at the time of admission. He developed metastatic deposits to the brain in both frontal lobes and died of intracerebral hemorrhage due to angiosarcomatous metastases into the brain [31]. Wong et al. reported a case of MPNST of the right radial nerve that extended from the elbow to the brachial plexus in a 59-year-old woman with no underlying NF1. The tumor was preceded 10 years earlier by inflammatory neuropathy followed by traumatic neuroma. The tumor and the triceps muscle were removed en-bloc, followed by radiotherapy. Follow-up for eight months showed no evidence of recurrence or metastases [32].

An unusual case of epithelioid MPNST of the left radial nerve with lung metastases in a 65-year-old male without any of the stigmata of NF1 was reported by Honma et al. The tumor posed a diagnostic challenge featuring highly cellular areas of polygonal or rounded cells, resembling lymphoma or melanoma, while the metastatic tumors revealed cord formation or rows, resembling carcinoma. Ultrastructural studies confirmed the nature of the tumor as an epithelioid variant of MPNST [33].

WHO defines neurofibromatosis type 1 (NF1), as an autosomal dominant disorder characterized by cafe-au-lait spots, axillary and inguinal freckling, Lisch nodules of the iris, and multiple neurofibromas. Cutaneous neurofibromas are typically benign, whereas plexiform neurofibromas have a risk of becoming malignant [34]. In their review of the risk of development of MPNSTs in patients with NF1, Evans et al. collected patients who had both NF1 and MPNST from two sources in North West England with a population of 4.1 million in a 13-year period (1984-1996). They found out that individuals with NF1 have a lifetime risk of 8-13% for the development of MPNST [35].

MPNST is a highly aggressive neoplasm, with a poor survival. There had been several series from major medical center looking into the treatment options of this neoplasm, including surgical resection, and its relationship to outcome and survival. In one series from the Mayo Clinic reporting outcomes of 25-year (1985-2010) experience treating MPNSTs, there were 175 patients, 57 of them (33%) had NF1. Surgical resection was performed in 166 patients (95%). Of these, 10 (6%) patients were treated with adjuvant chemotherapy alone, 69 (42%) patients were treated with adjuvant radiation alone, and 37 (22%) with combination adjuvant chemotherapy and radiation. Nine (5%) patients were lost to follow-up after surgery. The local recurrence rate was 22%, and 5- and 10-year disease-specific survival (DSS) was 60% and 45%, respectively. On multivariant analysis, size ≥ 5 cm, local recurrence, high tumor grade, and truncal location were poor prognostic indicators for DSS [36].

In a study from the French Sarcoma Group detailing their experience in the management and the outcome of 353 patients with MPNSTs, 37% were with NF1 and 59% were sporadic; it was observed that 294 patients (83.2%) underwent a curative intent surgery. Among them, 60 patients (21%) had neoadjuvant therapy (mainly chemotherapy) and 173 (59%) had had adjuvant treatment (mainly radiotherapy). In multivariate analysis, poor prognosis factors for overall survival (OS) were high grade, deep location, locally advanced stage at diagnosis, and macroscopically incomplete resection. NF1 status was not negatively prognostic, except in recurrence or metastatic setting [37].

In a recent study from the Netherlands, a nationwide cohort study on treatment and survival in 629 patients with MPNSTs, including 35 retroperitoneal, were selected out of 784 cases with definitive pathological diagnosis. Analysis of these patients showed that in surgically resected patients (88.1%), radiotherapy and chemotherapy were administered in 44.2% and 6.7%, respectively. In nonretroperitoneal tumors, old age above 60 years, presence of NF1, size more than 5 cm, and deep-seated tumors were independently associated with worse survival. Radiotherapy and chemotherapy administrations were not associated with survival [38].

The left elbow mass in our patient was diagnosed initially as neurofibroma on a tru-cut core biopsy done at a peripheral hospital. After the excision of the tumor, it was found to be a MPNST. Such discrepancy in the preoperative diagnosis of peripheral nerve sheath tumors (PNST) including benign PNSTs (BPNSTs) and malignant PNSTs (MPNSTs) with the postoperative diagnosis had been addressed by Graham et al. in their study of oncologic accuracy of image-guided percutaneous core needle biopsy (IGCNBx) of PNSTs at a sarcoma center. They collected 78 cases of PNSTs with both IGCNBx and postresection surgical pathology. There were 59 cases of BPNSTs (76%) and 19 cases of MPNSTs (24%). IGCNBx demonstrating schwannoma or MPNST were 100% accurate in determining malignancy. On the other hand, out of 12 neurofibromas diagnosed on IGCNBx, four (33%) proved to be MPNSTs on postresection surgical pathology [39]. This discrepancy is likely because of the heterogeneous nature of neurofibromas. Also, MPNST can be either low grade (15%) or high grade (85%). The term low-grade MPNST refers to less patently anaplastic tumors arising in transition from a neurofibroma precursor [40].

We reported earlier from our hospital a 23-year-old male patient without the stigmata of NF1 who had MPNST with extensive osteosarcomatous and chondrosarcomatous pluridirectional differentiation arising in the back at the left side of the upper lumbar spine, which was diagnosed clinically as a lipoma. The patient developed pulmonary metastases within nine months [41]. Another case reported from our hospital was related to a triton tumor of the right seventh intercostal nerve in a 28-year-old male with NF1 [42]. In both cases, no glandular differentiation was observed. Furthermore, we reported a series of three mediastinal triton tumors, none of them had a glandular component [43].

The differential diagnosis of MTT includes rhabdomyosarcoma and leiomyosarcoma. The presence of a few scattered S-100 protein-positive cells in foci of tumor away from those featuring rhabdomyoblastic differentiation is a useful clue in distinguishing MTT from rhabdomyosarcoma [25]. Leiomyosarcomas lack the eosinophilic strap-shaped cells with cytoplasmic cross striations and they lack immunoreactivity for myogenin [25].

The differential diagnosis of MPNST with glandular differentiation is synovial sarcoma. Glandular MPNST often arises from a neurofibroma or from a nerve while synovial sarcoma does not. Goblet cells and neuroendocrine cells can be frequently encountered in glandular MPNSTs but not in synovial sarcomas. Cytokeratin and EMA positivity are observed in the glandular component only on glandular MPNSTs, while they are positive in both glandular and nonglandular components of synovial sarcomas [25].

In conclusion, we encountered a case of MTT with malignant glandular component, the third case on record. The tumor was excised surgically but it recurred. Adjuvant chemotherapy and radiation therapy were administered with marked reduction in the size of the recurrent tumor. The patient had been disease-free for 10 years, which contrasted with previously reported devastatingly poor prognosis of these tumors.

## Figures and Tables

**Table 1:** Reported cases of triton tumor with glandular differentiation.

No.	Authors year [reference]	Age/sex	NF1	Location	Divergent differentiation components in MPNST	Treatment and outcome
1	Després et al. 1973 [5]	38/F	Yes	Sciatic nerve	RMS and benign glands	Surgery; recurred at 13 months; DOD at 19 months
2	Warner et al. 1983 [6]	29/M	Yes	Thigh	RMS and benign glands	Surgery; no follow-up data available
3	Daimaru et al. 1984 [7]	27/F	Yes	Buttock	RMS and benign glands	Surgery; DOD after 4 months
4	Christensen et al. 1988 [8]	NAD	NAD	NAD	RMS and benign glands	NAD
5	Christensen et al. 1988 [8]	NAD	NAD	NAD	RMS, osteosarcoma, and benign glands	NAD
6	Ho and Che 1989 [9]	14/F	Yes	Left sciatic nerve	RMS and benign glands of intestinal type epithelium	Surgical excision of the tumor (30 × 14 × 13 cm); no follow-up data available
7	Wong et al. 1991 [10]	39/M	No	Thorax, overlying T1-T4	RMS and malignant glands	Surgery, radiotherapy, and chemotherapy; recurred at 1 year; DOD at 15 months
8	Rose et al. 1992 [11]	34/M	Yes	Brachial plexus	RMS and glands with moderate nuclear atypia	Surgery, radiotherapy, and chemotherapy; recurred at 14 months; DOD at 18 months
9	Woodruff and Christensen 1993 [12]	20/F	Yes	Right side of neck, associated with plexiform neurofibroma	RMS, chondrosarcoma, osteosarcoma, and benign glands	Subtotal surgical resection; DOD after 3 months
10	Ordonez 1998 [13]	28/M	No	Right lumbar area, overlying L5	RMS and glands lined by a single layer of columnar cells or by multiple layers of cuboidal cells having marked nuclear atypia	Surgery, radiotherapy, and chemotherapy (Adriamycin); DOD after 33 months
11	Karpuz et al. 2000 [14]	52/M	No	Chest wall	RMS and benign glands	Surgery; recurred after 5 years
12	Huang et al. 2003 [15]	24/M	No	Thigh	RMS and malignant glands	Surgery; lung metastasis after 5 months; DOD after 6 months
13	Guo et al. 2012 [16]	79/M	NAD	Thigh	RMS, glands, lipoblasts, and ganglion cells	Surgery, radiotherapy, and chemotherapy; recurred after10 months, without metastasis
14	Thway et al. 2015 [17]	40/M	No	Mediastinum	RMS and benign glands	Surgery; brain metastasis after 15 months; transferred for palliation at 24 months
15	AlAli and Amr 2020 current case	26/M	No	Elbow, radial nerve	RMS and malignant glands	Surgery, radiotherapy, and chemotherapy; no recurrence or metastases for 10 years

Abbreviations: RMS: rhabdomyosarcomatous; DOD: dead of disease; NAD: no available data.

## Data Availability

The case report data used to support the findings of this study are included within the article.
